# Supplementary material for: Are carbon emissions trading and green financial instruments synergistic? -Comprehensive quantitative research based on content analysis
Source: PLoS One. 2024 Mar 7;19(3):e0298601. doi: 10.1371/journal.pone.0298601 (PMC10919601; doi:10.1371/journal.pone.0298601)
Supplement: S1 Data — (ZIP) [file pone.0298601.s001.zip › Data/地级市Z_F工作报告.zip!/╜¡╬≈/╥╦┤║╩╨/└┤╫╘╣1⁄2╓┌║┼í╛┬φ┐╦╩2╛▌═°í┐_2005.docx]

各位代表：

现在，我代表市人民政府向大会作政府工作报告，请予审议，并请市政协委员和列席会议的同志提出意见。

一、2004年政府工作回顾

过去的一年，是宜春经济和社会发展进程中极不平凡的一年。面对筹办农运会和加快发展的双重压力，市政府在省委、省政府和市委的正确领导下，坚持以邓小平理论和“三个代表”重要思想为指导，深入贯彻党的十六届三中、四中全会和省委十一届七次全会精神，认真落实国家宏观调控政策，全面树立科学发展观，围绕年初确定的工作目标，克难奋进，开拓创新，八项重点工作全面推进，九大工程顺利实施，十件实事基本完成，农运会取得圆满成功，经济社会保持快速协调发展。

经济发展步入快速增长期，主要指标增幅再创新高。继2003年结束多年低位徘徊局面后，经济发展速度进一步加快，主要经济指标提前一年完成“十五”计划目标。全市生产总值289.5亿元，增长13%，是近10年最好水平。财政总收入25.33亿元，增长22.7%，创近9年最高增幅，其中，地方财政收入15.46亿元，增长17.5%，创近7年最高增幅。财政总收入超亿元县市达8个，丰城市超6亿元；超千万元乡镇（街道）24个，比上年增加11个。全社会固定资产投资120亿元，是“十五”计划目标的1.6倍。丰城、樟树市评为全省经济发展先进县市。

工业经济增势强劲，主导地位更趋明显。全市规模以上工业企业完成增加值42.8亿元，增长30.8%；销售收入突破百亿元大关，达到119.7亿元，增长41.5%；利税总额11.65亿元，增长14.4%。工业用电量达11.1亿千瓦时，增长24.7%。工业经济综合效益指数为108.7%，提高3.5个百分点。奉新、上高县被评为全省工业发展先进县。三次产业比重为30.6：38.5：30.9，工业主导地位更趋明显，基本形成了医药、食品加工、建材、机械电子和电力能源等支柱产业。丰电公司、樟树四特集团等大中型骨干企业发挥了重要支撑作用，其中四特集团成为全市首户纳税过亿元的地方工业企业。工业园区拉动作用明显，新增工业固定资产投资45.9亿元，新增投产企业154家，园区企业实现销售收入79.4亿元，实现税金4.5亿元，安置就业8万多人。

农业生产形势喜人，农村工作再创佳绩。中央和省加强农业生产及减轻农业税费等各项政策措施全部落实到位，全市发放良种补贴6971.9万元、粮食直补8209.4万元，降低农业税税率、取消农业特产税，农民直接受益1.6亿元。农民生产积极性空前高涨，粮食播面865.5万亩，增长15.6%；粮食总产316万吨，增长21%，创历史最高水平。丰城市荣获“全国十大粮食生产标兵县（市）”称号，樟树市评为“全国粮食生产先进县（市）”。结构调整步伐加快，农业产业化稳步推进。已建立省级无公害农产品基地55个，创绿色食品品牌20个，16个有机农产品通过国家认证，万载县茭湖乡成为全国第一个乡域整体实施有机农业生产的乡镇，高安市评为“全国无公害蔬菜基地县（市）”，宜丰县荣获“全国食品工业强县”称号，靖安县获“中国椪柑之乡”称号；十大农业主导产业实现综合产值36.9亿元，税收9800万元，分别增长74.3%和20.5%。土地市场秩序治理整顿通过省政府验收，市国土资源局被国家人事部和国土资源部评为先进单位。林业重点工程进展顺利，实施退耕还林36.4万亩。

开放型经济取得突破，投资多元化初步确立。开放型经济实现“四个突破”：实际引进市外资金突破100亿元，达到133.1亿元，增长42.7%；实际利用外资突破1亿美元，达到1.29亿美元，增长54.1%；批准合同外资突破2亿美元，达到2.3亿美元，增长79.7%；外贸出口突破5000万美元，达到5970万美元，增长79.4%。对外经济技术合作取得新进展，上高县成为全省首批外派劳务基地县，宜丰县奋发竹业有限公司开创我市工业企业到国外投资先河。宜春检验检疫局评为全国质量监督检验检疫工作先进单位。民营经济不断壮大，全市个私企业达8万余户，注册资金92.4亿元，分别增长8%和48%；民营经济上交税金12.88亿元，占财政总收入的50.9%，比上年提高9个百分点。金融对地方经济支持力度进一步加大，金融机构各项贷款余额214.7亿元，增长15.7%，增量居全省第三。

基础设施日益完善，城乡面貌显著改观。公路建设实现新突破，新建水泥（油）路1482公里，其中农村公路建设规模超历史，竣工里程达1317公里，新增通水泥（油）路乡镇11个、行政村258个；昌金高速公路的建成，结束了宜春中心城区不通高速公路的历史；万载——宜春和宜春中心城区北绕城公路的建成，结束了我市没有一级公路的历史。电力、水利工程建设力度加大，丰电二期扩建工程进展顺利，赣抚大堤、黄金水库、樟树岭水库除险加固及城市防洪等重点水利工程建设稳步推进。城市化进程加快，城市化率为31%，提高2个百分点。宜春中心城区基础设施建设力度加大，投入资金8.67亿元，增长39.9%，完成了宜阳大桥、宜春大道、袁河西路、城区沥青路面铺设、小街小巷改造等一批重点工程，袁山公园、状元洲公园、迎宾公园基本建成，袁河水厂主体工程进展顺利，污水处理厂建设开始启动。县市城区面貌发生显著变化，建设了一批精品项目。农村沼气工程取得历史性突破，新建沼气池1.1万个，是上年的3.5倍。旧村改造全面推进，2316个村开展规划编制，255个村进行试点。文明村镇建设试点进展顺利，有14个镇、24个村开展了试点。

筹办农运圆满成功，谱写了宜春历史的辉煌篇章。在党中央、国务院和省委、省政府的亲切关怀和高度重视下，在社会各界的支持帮助下，全市上下围绕“成功看安全，圆满看接待，品牌看表演”的总体目标，克服种种困难，挑战巨大压力，用有限的资源和条件，夺取了筹办农运会的全面胜利。宜春中心城区开展了声势浩大的迎农运、创“三城”活动，一举获得省级文明城、卫生城、园林城称号；320国道境内沿线全面进行了环境整治，面貌焕然一新；开幕式大型文体表演气势恢宏，竞赛工作井然有序，接待工作周密细致，安全保卫万无一失，后勤保障及时到位。整个筹办工作不仅得到了党和国家领导人的充分肯定，受到了省委、省政府的通令嘉奖，而且在这次严峻的考验中，铸就了我们宜春“坚韧不拔、不畏艰难，事在人为、负重奋进，众志成城、敢创一流”的精神。

人民生活水平稳步提高，社会保障体系不断完善。城乡居民收入大幅增加，城镇居民人均可支配收入7132元，增长9.5%；农民人均纯收入3164元，增长17.7%。城镇居民人均住房建筑面积达到21.7平方米，农村居民人均居住面积达到35平方米。社会消费品零售总额128.2亿元，增长12.1%。消费结构发生变化，城镇居民恩格尔系数降为39%，假日旅游、通信电子产品、住房等成为新的消费热点，移动电话用户71万户，增长24.6%；固定电话用户75万户，增长21%。就业再就业工作成效显著，城镇新增就业人员4.3万人，下岗失业职工再就业2.97万人，劳务输出64.5万人，城镇登记失业率控制在3.5%以内，市劳动和社会保障局、袁州区政府再就业工作受到省政府表彰。社会保障体系框架基本确立，22.5万人参加基本养老保险，确保了企业离退休职工养老金按时足额发放；24.3万人参加职工失业保险；基本医疗保险全面启动并正常运转，参保人员23.2万人。全市劳动和社会保障工作名列全省第一。12.3万人纳入城市居民最低生活保障，实现了动态管理下的应保尽保；12万名农村特困群众得到了及时救助，57万名因旱因灾的农村群众和4万余名移民生活得到了妥善安排。

社会事业不断进步，精神文明建设得到加强。科技创新能力进一步提高，争取省级以上科技项目70项，其中国家级14项，发展省级民营科技企业8家。教育事业开创新局面，“两基”迎省复查年检工作受到省政府表彰，铜鼓县山区寄宿制办学模式在全国、全省推介，宜春中学、丰城中学等一批优质高中新校区基本建成，高考二本上线总人数连续七年列全省第一，宜春学院面向全国27个省市区招生，在校生达1.4万余人。医疗救治、疾病预防控制、突发公共卫生事件应急体系建设日趋完善，县、乡、村三级公共卫生信息网络建设全面完成。文化事业蓬勃发展，成功举办市第二届艺术节，修复了袁州古天文台。体育工作取得历史性突破，雅典奥运会上杨文军实现了宜春奥运金牌零的突破，第五届全国农运会上我市获11枚金牌。旅游产业逐步壮大，重点景区景点建设步伐加快，省级风景名胜区增至5个，靖安三爪仑被国家旅游局授予3A级旅游景区，全市实现旅游总收入13.62亿元，增长33%。广播电视和新闻出版事业全面发展，外宣工作进一步加强。人口和计划生育工作进入全省先进行列，人口自然增长率控制在7.58‰以内，农村部分计划生育家庭奖励扶助制度试点工作成效明显，成为全国计划生育工作会上唯一作试点工作典型发言的地级市。群众性精神文明创建活动广泛开展，思想道德建设不断加强，高安市八景镇和袁州区温汤镇评为全国文明村镇工作先进单位。

各项改革进展顺利，安全稳定得到有效维护。市、区管理体制进一步理顺，城市综合管理体制基本确立。市与县、县与乡财政管理体制进一步完善，市县乡财政分配关系更加明晰。农村信用社改革、卫生体制改革稳步推进，乡镇卫生院全部上划县管，樟树市新型农村合作医疗试点工作运行平稳。宜丰县事业单位人事制度改革取得阶段性成果，铜鼓县林业产权制度改革试点进展顺利，奉新县产权交易和经营性公房管理等做法得到了中纪委、中组部的肯定。国有企业改革扎实推进，宜齿公司改制基本结束，江麻公司改制进展顺利，市汽运公司成功收购萍乡汽运公司，江特公司成为全省首家进入上市辅导期的县属企业。城市公用事业改革取得突破，市供水公司股权转让顺利完成。安全生产形势保持平稳，杜绝了特大生产安全事故的发生。社会治安防控工作机制逐步完善，人民内部矛盾得到妥善处理。市场经济秩序进一步规范。公民法制观念、道德观念和诚信观念得到增强。

政府廉政建设和依法行政意识进一步增强。开展了发展环境创优年活动，经济发展环境进一步优化。以贯彻《行政许可法》为契机，切实加强了对行政权力的监督，规范执法行为，清理并取消不属于行政许可的项目119项；对土地等有限资源的开发利用和公共自然资源的配置，依法采取了招标、拍卖、挂牌出让等方式；对事关人民群众切身利益的收费项目出台和价格调整，全部进行听证；对损害群众利益问题进行了专项整治。民主法制建设进一步加强，自觉接受人大工作监督、法律监督和政协民主监督，人大代表建议、政协委员提案206件全部办复。国家安全、人民防空、民兵预备役、民族宗教、审计、统计、人事编制、信访、扶贫、监察、人民调解、地震、气象、档案、史志、社会科学、老龄和妇女儿童工作等各项事业都有新进步。

各位代表，过去的一年，是全市经济取得突破性进展，综合实力不断提升，人民群众生活大为提高的一年；是城乡面貌日新月异，发展环境明显改观，对外形象显著提升的一年；是各项社会事业全面发展，社会主义精神文明和民主法制建设取得新进步的一年。成绩的取得，是省委、省政府和市委正确领导的结果，是市人大常委会依法监督、市政协民主监督的结果，是全市人民辛勤劳动和团结奋斗的结果。在此，我谨代表市人民政府，向全市人民致以崇高的敬意！向参与、支持和关心宜春发展的广大工人、农民、知识分子、公安干警、驻宜人民解放军、消防官兵、武警部队、中央和省驻宜单位、各民主党派、工商联、无党派人士、社会团体、离退休老同志和海内外朋友，表示衷心感谢！

在肯定成绩的同时，我们也清醒地看到经济社会发展中还存在不少困难和问题，主要是：经济总量小，人均水平低，结构性矛盾较为突出；招商引资项目技术含量、产品档次总体偏低，大企业不多；传统农业比重仍偏高，农业产业化水平较低；社会保障体系还不健全，部分群众生活比较困难，就业压力仍然很大；因体制转换或工作不到位引发的社会不稳定现象仍时有发生；安全隐患尚未得到彻底消除；综合改革的力度不够大，政府的工作方式、工作作风、服务质量有待进一步改进和提高，等等。对上述问题，我们将高度重视，认真对待，在今后的工作中切实加以解决。

二、2005年政府工作的目标要求和工作重点

2005年是贯彻落实科学发展观，深化改革、扩大开放、加快发展的关键一年，也是在新的起点上加快发展的重要一年，做好今年的政府工作，对于巩固经济社会发展的良好态势、衔接“十一五”发展、全面建设小康宜春具有十分重要的意义。

综合分析宏观经济和区域经济发展的形势，我们将面临重要的战略机遇期。从全球范围看，世界经济继续复苏，经济全球化和贸易投资自由化趋势增强。从全国范围看，国家强调促进区域经济协调发展，区域经济合作加速推进，内地与香港、澳门建立了更紧密的经贸关系，长三角、珠三角和闽三角加快了产业转移步伐，有利于我们发挥比较优势，在错位竞争中更多地承接沿海地区的产业转移。从全省范围看，我省经济发展进入了平稳快速增长的新阶段，经济结构进入了转换升级的新层面，经济运行进入了更加注重质量和效益的新轨道，江西崛起的大势将进一步坚定我们加快发展的信心和决心。从全市范围看，继2003年呈现转折性变化之后，去年全市经济社会发展又迈上了新的台阶，特别是成功举办第五届全国农运会，显著提升了宜春的知名度和影响力，全市上下干事业、求发展的积极性空前高涨，宜春正迎来一个千载难逢的发展黄金期。我们一定要增强紧迫感和责任感，以超常的胆识抢抓机遇，以超常的气魄扩大开放，以超常的思维加快发展。

根据中央经济工作会议和省委十一届八次全会精神，今年政府工作的总体要求是：以邓小平理论和“三个代表”重要思想为指导，牢固树立科学发展观，紧扣第一要务，弘扬农运精神，突出改革主线，倡导创业创新，全力推进大开放主战略和“心圈廊”发展构想，加快“三化”进程，着力提升经济社会发展质量和水平，做大市本级，激活县市区，关注民生，维护稳定，努力开创建设赣西经济中心城市与和谐平安宜春的新局面。全市经济社会发展主要预期目标是：生产总值增长11%，力争13%；财政收入增长13%，力争17%，其中地方收入增长11%，力争15%；农民人均纯收入增加200元，城镇居民人均可支配收入增加400元；全社会固定资产投资增长20%，力争25%；实际利用境外资金增长55%，力争85%，实际引进市外资金增长27.8%，外贸出口增长67%，力争100%；社会消费品零售总额增长11%；城镇登记失业率控制在4.5%以内，人口自然增长率控制在8‰以内，城市化率达到32%。

做好今年的政府工作，必须把握全局，突出重点，求真务实，坚持以科学发展观统筹经济社会发展全局，努力把对科学发展观的认识成果转化为促进发展的正确思路，转化为实现全面、协调、可持续发展的体制机制，转化为领导改革开放和现代化建设的实际能力。具体工作中，坚持和把握好五项原则：一是坚持加速推进经济增长方式的转变；二是坚持统筹城乡发展；三是坚持加强生态建设和环境保护；四是坚持促进社会主义物质文明、政治文明、精神文明协调发展；五是坚持把造福群众作为政府工作的不懈追求。

促进全市经济更快更好的发展，必须紧紧抓住涉及经济社会发展全局的重大问题，围绕开放型经济这一主体，突出改革创新这一主线，抓住优化环境这一关键，强化发展保障这一根本，扎扎实实做好以下几方面工作。

(一)围绕一个主体，坚持大开放主战略，培育支柱，提升经济发展总体水平

坚持以开放型经济为主体，必须突出工业化核心战略，以招商引资为总抓手，加快工业园区建设，提高全市经济的外向度。大力开展“质量兴市”和“名牌兴企”工程，通过开放带动，培育壮大一批骨干龙头企业，做大做强一批优势园区，加速推进产业集聚，培植支柱产业，以有效利用国际经济规则为手段，促进全市经济总量增长、质量提高、结构优化。

做大做强工业园区。按照土地集约化经营、产业链式化延伸、企业集群式组合、资源循环式利用的要求，抓好建设、管理和服务三个环节，着力提升园区开发水平，增强园区对产业集聚、集群发展的吸引力和服务能力。加快硬件建设，重点抓好园区水、电、网络的配套和服务，7月份前完成市经济技术开发区二期“三通一平”。适度提高入园门槛，建立入园项目评价体系，将投资规模、投资强度、单位面积上缴税金、环境影响等作为项目评价指标，走科技型和环保型园区建设之路。梳理入园项目，合理利用和珍惜园区土地，清理整顿“圈而不建”、“圈大建小”以及“半拉子”工程。强化配套服务，适应产业集群发展需要，加快物流、研发、人才、金融等公共服务平台建设，建立入园项目跟踪服务制度，及时协调解决有关问题，努力提高开工率、投产率、达产达标率。力争全市工业园区新增固定资产投入50亿元，其中亿元以上项目10个以上；新增投产企业200家以上，新增销售收入40亿元，上交税金6亿元，安排就业人员10万人。其中市经济技术开发区入园企业总量今年要达到100家以上。

调整招商引资思路。明确定位、转变方式、完善机制，推动招商引资由游动分散型向固定网点型、由自然接受型向产业聚集型转变。注重引进大企业、大产业，盯准产业中的龙头企业、核心企业和带动作用明显的大企业，力争引进国内外500强企业、上市公司等知名企业。突出优势招商，充分挖掘现有产业优势、资源优势，尤其是劳动力资源优势，以建设轻工业城为目标，大力引进纺织服装、医药食品、机械电子等产业，以及汽摩配、鞋业、锁具制造、反光材料等产业关联度大的项目。突出重点区域招商，国内紧联“长珠闽”，境外突破“港澳台”，国外盯住“日韩美”，在发挥好驻外办事处作用的基础上，增设东莞、泉州两个固定招商点，重点引进当地有转移意向的外资企业；加大在温州的招商力度，继续发挥好300名挂职干部的作用，做到与温州企业界的联系不断，感情不淡。今年，在境外至少组织1－2次招商引资推介活动。

提高经济外向度。高度重视外经外贸工作，支持有条件的企业到境外投资、兴办实体或设立销售点。注重培育有国际竞争力的出口产品，大力支持企业申请国际质量标准体系、环保体系、安全体系和产品认证，引导、帮助出口企业申请原产地保护和到境外注册商标。充分利用国内、国外两种资源、两个市场，进一步扩大出口。落实国务院、省政府出口激励措施，做好出口退税工作。做好建立海关的各项前期准备工作，引进出口保险机构并落实扶助措施。积极争取批准增加市本级和丰城市两个境外劳务输出基地，支持具备条件的企业到境外开展工程承包业务。

（二）突出一条主线，着力推进各项改革，勇于创新，全面激活发展的内生活力和动力

牢牢把住改革这条主线，将改革同贯彻科学发展观、加强和改善宏观调控紧密结合起来，努力革除体制障碍，消除机制弊端，力求在一些重要领域和关键环节上实现重大突破，提高经济可持续发展能力。

深化国有资产管理体制和国有企业改革。坚持政府公共管理职能和国有资产出资人职能分开，抓紧组建和完善国有资产监管机构，用三年左右时间，探索建立起新的国有资产监管体制基本框架。以现代产权制度为核心，积极推行国有企业股份制改造，大力发展混合所有制经济，完善国有资本有进有退、合理流动的机制。以明晰产权为重点，加快市直国有企业资产优化重组，尽快完成宜春齿轮厂的破产扫尾工作，抓紧江麻公司、市建总公司、长青厂改制和汽运公司股份制改造。年内基本完成市、县属国有、集体企业改革。加快资本、土地、技术、产权等要素资源整合，在盘活城区机关单位存量土地、推进公共财政体系建设等方面重点研究，制定措施。在改革中注重操作的科学性，坚持一切从实际出发，因地制宜，把改革的力度、发展的速度和社会可承受的程度统一起来，及时化解各种矛盾，同时规范改制程序，确保国有资产不流失，确保职工合法权益得到维护。

切实转变政府职能。把政府经济管理职能转到主要为市场主体服务和创造良好发展环境上来，全力打造服务型政府。强化政府社会管理和公共服务职能，健全新形势下处理人民内部矛盾和社会矛盾的有效机制、社会治安综合治理机制和城乡社区管理机制，加快公共基础设施和公共服务系统建设，建立健全预警和应急机制。大力发展规范性的行业协会和社会中介组织，有效发挥市场在资源配置中的基础性作用，改变政府在微观经济活动中干预过多的现象。改革和理顺行政管理体制，加强对各级行政机关管理、收费、罚没权的制约和监督，进一步清理整顿收费项目和标准，以行政许可项目进服务中心办证大厅为切入点，规范行政许可行为。规范部门行政处罚自由裁量权，相对集中行政处罚权，推进城市管理领域的综合执法。认真抓好市政府职能机构调整，理顺部门分工，防止和纠正部门本位主义和地方保护主义。

全力推进事业单位改革。按照政事分开的原则，分类定位、分开管理、分别改革。对行政管理类事业单位，加快清理、归并、精简和规范，重新界定行政职能，经费由财政供给，严禁以收抵支、以罚代养；对社会公益类事业单位，调整布局结构，优化资源配置，增加政府投入，提高服务水平；对经营开发类事业单位，按照市场化、社会化、产业化方向，加快转企步伐，引入市场竞争机制，吸收多元化投资，增强发展活力。推进事业单位用人制度和分配制度改革。市直事业单位改革要带头突破，重点抓好自收自支事业单位的“国退民进”工作，通过开展深入细致的调查研究，理顺各个方面、各个层面，尤其是深层次的利益关系，制定指导性强、操作性强的改革方案，尽快实施。

（三）抓住一个关键，切实创优发展环境，提高效能，大力营造干事创业的浓厚氛围

紧紧抓住优化环境这个关键，以打造效能政府为切入点，深入开展“规范服务效能年”活动，规范各级政府的政务行为，提高行政效能，不断优化民营经济大发展的环境、做旺城市人气的环境和“三农”工作的发展环境，努力营造干事创业、富民兴市的浓厚氛围。

优化民营经济大发展的环境。在全市上下进一步培育创业、和谐的社会文化和社会环境，以创业带动就业，以创业走向富裕。着力引导一大批市民、农民走自主创业之路，扶持一大批小老板在尽可能短的时间内由小变大、由弱变强，成为宜春经济一支最重要的发展力量。全面拓宽民营经济发展空间，按产业制定优惠政策，按行业提出扶持措施，帮助民营企业做大做强。强化民营经济的组织领导，把民营经济发展情况作为考核地方政绩的重要内容，成立强有力的民营经济发展组织机构，建立高效运转的工作机制，实行领导挂点帮扶企业制度，市县政府班子领导每人联系一户重点民营企业，挂点帮扶，两级经济管理部门和经济监督部门分别挂点帮扶一户民营企业，定目标、定任务、定奖惩，一年一考评，确保各项工作落到实处。

优化做旺城市人气的环境。在大力推进工业化的同时，把加快第三产业发展，作为聚旺城市人气的重要手段，着力建设赣西文化娱乐、职业教育、体育健身、旅游休闲、商贸物流等产业中心。在巩固提高九年义务教育的基础上，加快发展高中阶段教育，支持宜春学院和其他大中专院校做大做强；积极推进职业教育改革与发展，鼓励扶持民办教育发展，打造一批品牌民校。加大文化基础设施建设投入，积极发展高质量的文化娱乐，提高文化消费档次和水平。充分利用现有体育场馆，借助农运影响，更多地承接国际国内体育赛事及文化活动。整合旅游资源，加快以明月山、三爪仑为代表的自然生态景区，以铜鼓、万载为代表的红色旅游景区，以宝峰寺、仰山、洞山、百丈山为代表的宗教文化景区建设，做大做响禅宗文化和生态旅游品牌。创新思路，加快城市基础设施建设，提高城市管理水平。着力推进宜春中心城新城区建设，启动新城区路网工程，加快明月北路、高士北路延伸和钓台路、芦洲路等道路建设，完成袁河水厂、鼓楼路等重点工程，开工建设污水处理厂主体工程、煤气改气工程，争取宜春——安福公路改造开工。进一步严明土地、规划、房地产开发管理的政策法规，严厉打击违法建设行为，加大房地产市场调控力度，改善住房结构，进一步激活房地产市场。完善综合执法、社区创建、市民自治“三位一体”的城市管理模式，实现无缝隙覆盖，构建城市管理和创建的长效机制。以创新中国成立家生态园林城为总目标，分步骤逐步实现创新中国成立家文明城、卫生城、园林城、环保模范城、双拥模范城和中国旅游城市目标，年内争创“国家文明城”。继续加快丰城、樟树、高安中等城市建设步伐，尽快启动境内105国道一级公路建设，推进其它六个县城和省级重点镇建设，加大旧村改造和文明村镇建设力度，全面开展县市创建省级文明城、卫生城、园林城工作，力争三分之一的县城达到省级园林城市标准。

优化“三农”工作的发展环境。努力适应国内外市场的新变化，大力拓宽农民增收渠道和空间。提高农业综合生产能力，认真落实中央、省制定的各项惠农政策，突出抓好以优质稻为重点的粮食生产，增加播面和产量，提高粮食生产效益和商品率。实行最严格的耕地保护制度，严格控制农用地转非农建设用地。加快农业结构调整，大力发展高效经济作物和养殖业，继续拓展农业标准化示范区工作，扩大无公害农产品、绿色食品和有机农产品的生产规模，力争高效经济作物产值占种植业产值比重、养殖业产值占农业总产值比重分别提高5个百分点，无公害农产品、绿色食品和有机农产品个数增长30%以上。继续抓好十大主导产业，巩固扩大优质农产品基地，搞活农产品流通。开展速生丰产林基地建设，大力发展杨树产业。加强水利基础设施建设，实施好土地开发治理重点项目，改善农业生产条件。努力适应农村政策的新变化，积极化解取消农业税和农业特产税，以及全面推进林业产权制度改革后财政减收的压力，加强以发展个私经济为重点的财源建设，努力培植新的经济增长点。大力推进乡镇配套改革，精简人员，压缩开支，消化债务，确保乡村两级正常运转。努力适应农村工作内外部环境发生的深刻变化，适时调整乡村两级工作重心，切实改变过去主要依赖行政手段、重管理轻服务的工作模式，增强服务意识，引导乡镇干部把工作精力转到抓项目、跑市场、为农民提供各种服务上来，在新形势下争取有新的作为。加强基层组织建设，推进基层民主化。实行村务公开，民主管理，接受群众监督。

（四）强化一个根本，完善发展保障，统筹兼顾，努力打造和谐平安宜春

坚持以人为本，大力发展各项社会事业，协调各阶层利益，促进社会公平，激发社会活力，维护社会稳定，着力构建经济快速发展、社会安定祥和、人民安居乐业的和谐社会。

实施人才强市战略。尽快建立宜春市人才市场，健全服务体系，统筹人才资源整体开发，加大人才引进力度，优化人才资源配置。继续实施“6666”人才工程，重奖6名作出突出贡献的专家，选拔培养60名左右学科带头人，开发培养600名优秀后备人才，选拔组织6000名科技人才结对扶贫。依靠科技进步与创新，充分发挥科技人才的作用，增加科技投入，推进高科技研发、制造业信息化、新产品开发、粮食丰产科技等四大工程建设，提高科技成果转化率和对经济增长的贡献率。

推进社会事业发展。建立健全城乡一体的公共卫生体系和突发公共卫生事件应急机制，提升卫生服务能力和水平。完善农村新型合作医疗试点，建立健全合作医疗基金监督机制，扩大社区卫生服务覆盖面。强化公共卫生执法监督，规范医药价格和医疗收费，坚决纠正医药购销和医疗服务中的不正之风。发展广电产业，加快“村村通广播电视”步伐。完善人口与计划生育公共服务体系，稳定计划生育政策和低生育水平，提高人口素质，改善人口结构。做好老龄和残疾人工作。在继续抓好年鉴编撰工作的同时，抓好《宜春市志》的编修工作，力争两年内出版。

健全社会保障体系。建立城乡统筹的劳动力就业制度，启动“城乡青年职业技能培训计划”，充分发挥各类培训机构与社会办学力量的作用，广泛开展再就业和创业培训。继续实行积极的就业政策，千方百计拓宽就业渠道，增加就业岗位，确保全年城镇新增就业人员4.3万人、下岗失业人员再就业2.1万人。加强劳务输出和劳务协作，确保全年市外劳务输出60万人，力争达到65万人。完成外出务工人员基本情况调查，做好外出务工人员从事职业的调查登记，进一步抓好城乡劳动力资源的统筹管理及优化配置。依法扩大社会保障覆盖面，提高非公有制企业的参保率，继续抓好下岗失业人员养老保险的续保工作，制定完善灵活的就业人员参保办法，规范养老保险补缴政策，做好企业退休人员增加养老金工作。建立健全城乡困难群众社会救助体系，妥善解决其子女上学、看病就医等方面的实际问题，进一步提高农村“五保”人员集中供养率。

努力保持社会稳定。全面落实安全生产责任制，继续深入开展煤矿、花爆等11项专项整治，坚决打击非法生产。认真落实安全生产许可证制度，提高企业安全生产整体素质。加快建立安全生产长效管理机制，确保实现杜绝特大死亡事故、杜绝校园内死亡事故、减少重大死亡事故和一般死亡事故的目标。落实社会治安综合治理的各项措施，依法严厉打击各种刑事犯罪和黑恶势力，坚决扫除黄赌毒等社会丑恶现象，净化社会环境。落实减轻农民负担政策，妥善处理企业改革和城市建设中的各种矛盾和问题，继续做好清理拖欠工程款、民工工资和被征地农民补偿安置金工作。全面落实信访工作领导责任制，高度重视并切实做好信访和人民调解工作，及时化解社会矛盾，有效减少越级上访，依法妥善处置群体性、突发性事件，保持社会和谐稳定。

提高政府行政能力。按照科学执政、民主决策、依法行政的要求，推进政府管理创新，提高政府管理水平。全体政府工作人员特别是各级领导干部务必做到政治坚定、业务精通、清正廉洁、作风优良。带头维护宪法和法律的权威，严格按照法定权限和程序行使权力、履行职能，坚持科学民主决策，自觉接受群众监督。坚持全心全意为人民服务的根本宗旨，深入开展保持共产党员先进性教育活动，大力提高各级政府工作人员的能力素质。坚定不移地抓好党风廉政建设和反腐败工作，逐步建立健全教育、制度、监督并重的惩治和预防腐败体系，坚决纠正损害人民群众利益的不正之风。

各位代表，同志们，我们正处在创新体制、加快发展的关键时期，新的形势催人奋进，新的任务光荣艰巨。让我们紧密团结在以胡锦涛同志为总书记的党中央周围，高举邓小平理论伟大旗帜，全面贯彻“三个代表”重要思想，在省委、省政府和市委的正确领导下，同心同德，励精图治，开拓创新，扎实工作，为谱写宜春经济社会发展的新篇章而努力奋斗！
